# The prevalence and parameters of fabella and its association with medial meniscal tear in China: a retrospective study of 1011 knees

**DOI:** 10.1186/s12891-022-05092-4

**Published:** 2022-03-01

**Authors:** Jingyu Zhong, Guangcheng Zhang, Liping Si, Yangfan Hu, Yue Xing, Yaohua He, Weiwu Yao

**Affiliations:** 1 Department of Imaging, Tongren Hospital, Shanghai Jiao Tong University School of Medicine, No. 1111, Xianxia Road, 200336 Shanghai, China; 2 Department of Sports Medicine, Shanghai Jiao Tong University Affiliated Sixth People’s Hospital, No. 600 Yishan Road, 200233 Shanghai, China; 3 Department of Orthopedics, Shanghai Sixth People’s Hospital, Jinshan Branch, No. 147 Jiankang Road, 201500 Shanghai, China

**Keywords:** Fabella, Medial meniscal, Oblique popliteal ligament, Magnetic resonance imaging, Knee, Risk factor

## Abstract

**Background:**

Fabella is a sesamoid bone of knee that has potential biomechanical function. We aimed to examine the fabellar prevalence and parameters in Chinese population and test the hypothesis that fabellar presence and morphology were associated with meniscus tear or ligament injury.

**Methods:**

A total of 1011 knee magnetic resonance imaging scans from 979 patients with knee pain were analyzed retrospectively. The exclusion criteria are postsurgical scans, difficulty in fabella discrimination, conditions not suitable for measurement, and unsatisfied image. The fabellar presence and its parameters (length, width and thickness) were documented. The association between fabellar presence and meniscus tear or ligament injury were assessed by chi-square test, in all knees and subgroups (age, gender, side, lesion part). The correlation of fabellar presence and parameters with advancing age was assessed by Spearman correlation analysis. Odds ratios (ORs) and 95% confidence intervals (CIs) were calculated to investigate whether factors related with meniscus tear or ligament injury. Diagnostic performance of risk factors was assessed by receiver operating characteristic (ROC) analysis.

**Results:**

The overall prevalence of fabellae was 39.8% (402/1011 knees) and increased with the increasing age (r = 0.237, P < 0.001). The size of the fabellae differed according to genders, age, and presence of articulating grooves. Fabella presented more often in knees with medial meniscus (MM) tears (66.7% vs 33.8%; P < 0.001) with a multivariate OR of 2.960 (95% CI, 1.853–3.903). The association remained in all tear parts (anterior, middle, and posterior), and in younger (age < 50 years) and older patients (age ≥ 50 years). Age, fabellar length, width, length/thickness ratio and width/thickness ratio yielded an area under the ROC curve (AUC) of 0.604–0.766 to predict an MM tear. In combination with age, fabellar width and length/thickness ratio, the AUC was improved 0.791 (95% CI, 0.744–0.837), with a sensitivity of 73.0% and a specificity of 74.6%.

**Conclusion:**

The presence of fabellae, increased fabellar length and width as well as flatter fabellar morphology, are significantly associated with an increased risk for MM tear. These findings might aid clinicians in identifying patients at risk for a MM tear and informing them.

**Supplementary Information:**

The online version contains supplementary material available at 10.1186/s12891-022-05092-4.

## Background

The fabella is a small fibrocartilaginous body or sesamoid bone usually embedded in the tendon of the lateral head of the gastrocnemius muscle, articulated with the posterior surface of the lateral condyle of the femur [1, 2], and accompanied by oblique popliteal ligament (OPL) and fabellofibular ligament (FFL) [1–3]. The frequency of occurrence of fabellae ranges from 3.1 to 86.9% [4, 5]. In most cases, fabellae are ossified and can be seen radiologically, while cartilaginous fabellae can only be observed by magnetic resonance imaging (MRI) or anatomic methods [6, 7].

The fabella is usually believed to be a benign anatomical variant, although several studies have reported that the fabella is related to several disorders and diseases [5, 8–10]. Asian people, older people and knee osteoarthritis patients present with fabellae more frequently [3, 5, 7–9, 11, 12]. The size of the fabellae varies from a tiny dot to a body with a width of 22 mm [13]. Large grooves are more likely to be associated with articular grooves and are considered to have greater clinical significance [14]. Few studies on fabella have been published, with the majority focused on its size in a relatively small sample. Most of them were performed by roentgenogram methods, which have with a lower sensitivity in cartilage fabellae in comparison to those by MRI or anatomic methods, and were unable to describe the characteristics of the fabellae in detail. Moreover, most studies discussing the association between medical conditions and fabellae were case reports. To the best of our knowledge, no study has focused on the relationship between ligaments or meniscus injuries and fabellar presence. The purpose of this study was to examine the prevalence and parameters of the fabella in the Chinese population and to determine whether fabella is correlated with meniscus tears or ligament injuries. It was hypothesized that fabellar presence and parameters were related to meniscus tear or ligament injury and could not be identified as a simple normal anatomic variant.

## Methods

### Patients

**Fig.1:**
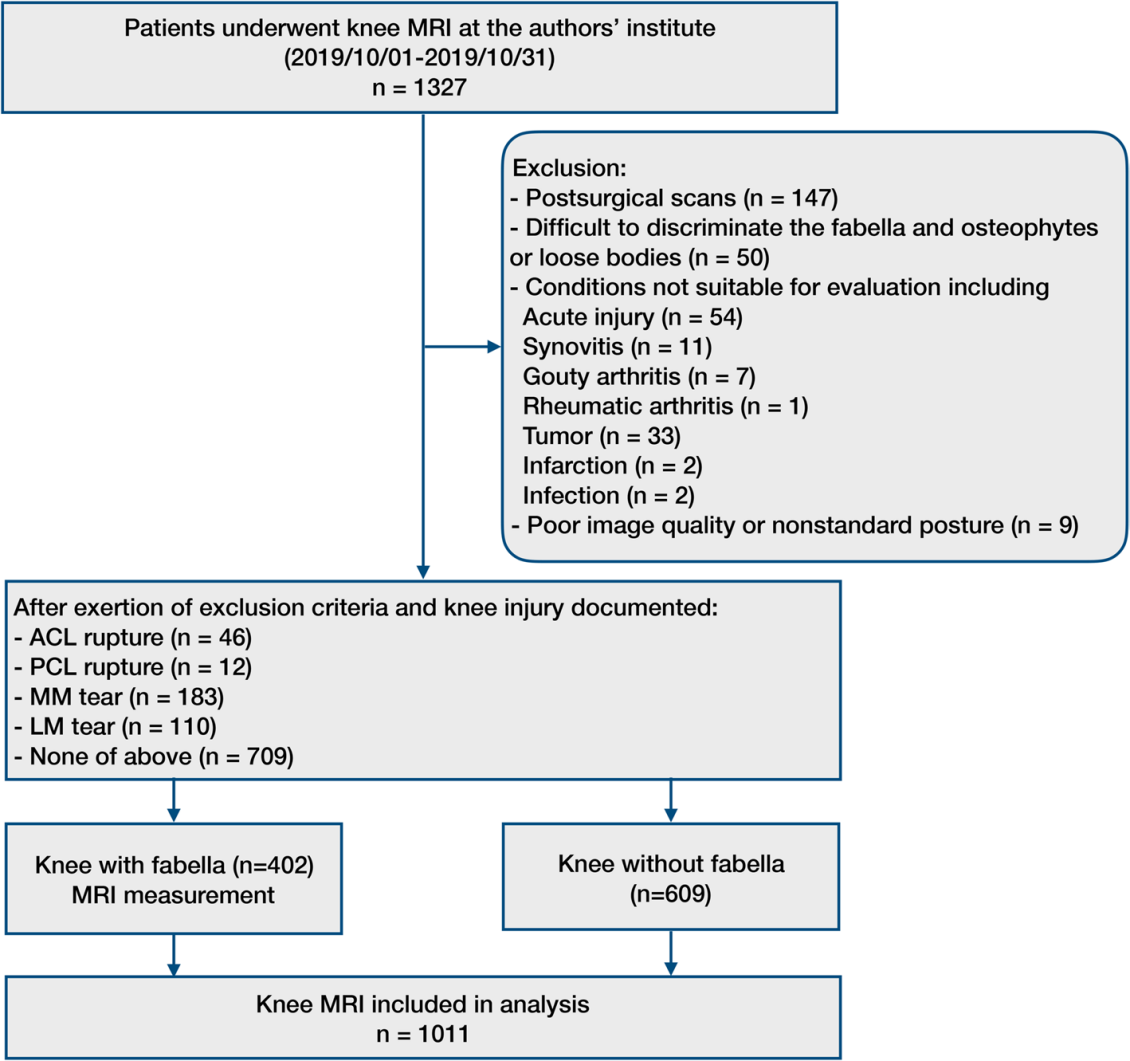
Flowchart and eligibility. *ACL* anterior cruciate ligament, *PCL* posterior cruciate ligament, *MM* medial meniscus, *LM* lateral meniscus, *MRI* magnetic resonance imaging

A retrospective review of consecutive patients who underwent MRI in our institution from October 1st to October 31st in 2019 was conducted (Fig. 1). Patients with knee pain were included. Patients were excluded before analysis as follows: (a) postsurgical scans; (b) difficulty discriminating the fabella and posterior osteophytes or loose bodies; (c) conditions not suitable for measurement, including knee malalignment, osteoarthritis, acute injury (acute knee dislocation, acute dislocated fracture), gouty arthritis, rheumatic arthritis, synovitis, tumors, etc.; and (d) poor image quality or nonstandard posture. Detailed exclusion criteria are available in the Appendix Material. Thus, a total of 1011 knee MRI scans were recruited for the final analysis. Clinical data, including age, sex, side of the knee, and related medical history, were retrieved from institutional database records. This study was approved by the ethics committee of Shanghai Jiao Tong University Affiliated Sixth People’s Hospital, and written informed consent was waived due to its retrospective observational nature. All methods and procedures were performed in accordance with the 1964 declaration of Helsinki and its later amendments.

### MRI measurements

MRI scans were performed on 3.0 T superconducting MRI scanners (Achieva, Philips Healthcare, Amsterdam, Netherlands; Ingenia, Philips Healthcare, Amsterdam, Netherlands; Magnetom Verio, Siemens Healthineers, Erlangen, Germany; Signa, GE Healthcare, Waukesha, WI, USA) with a standard protocol that included standard axial, coronal and sagittal imaging planes and a slice thicknesses of 3 to 5 mm for each plane. MRI sequences included T1-weighted imaging, T2-weighted imaging and/or proton density weighted imaging. All MRI scans were reviewed by a radiologist and an orthopedist independently using a reader (TViewU64, Winningsoft Inc., Shanghai, China). In our pilot study, we retrospectively included 100 consecutive knee MRIs in our department from October 1st to October 7th in 2019 (Additional file 1: Appendix Table A1). Only medial meniscal (MM) tears were found to be related to the presence of a fabella (*p* = 0.049). However, we decided to document patients with anterior cruciate ligament (ACL) rupture, posterior cruciate ligament (PCL) rupture, and lateral meniscal (LM) tears, who might be interested in orthopedists. The details of the pilot study are available in Appendix Table A1. In our formal study, the presence of ACL rupture, PCL rupture, LM tears and MM tears was documented. The final decision was reached with consensus after discussion in uncertain cases.

**Fig. 2:**
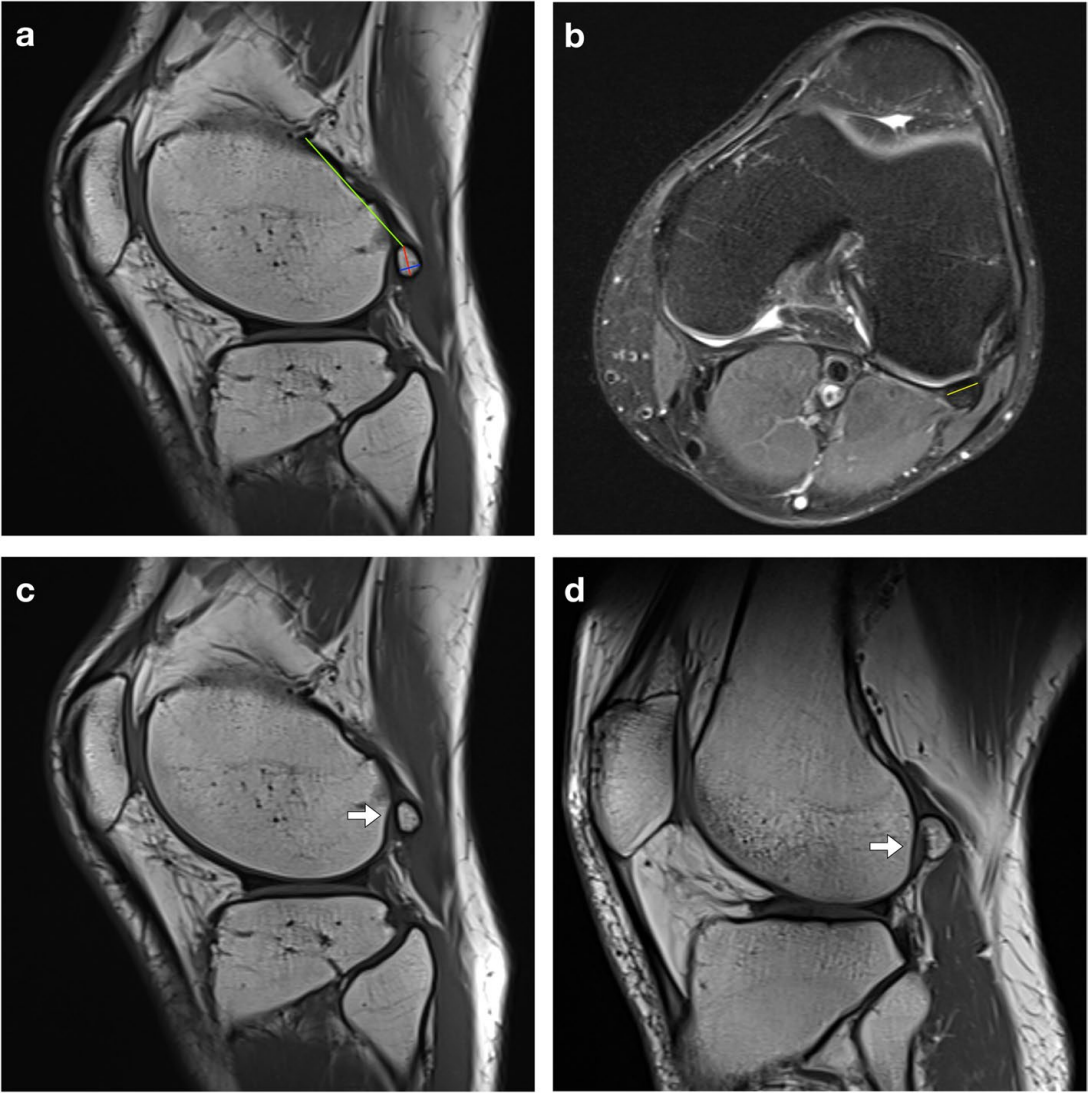
Magnetic resonance imaging (MRI) measurement and articulating groove. **a** Sagittal MRI of the knee shows the measurement of the maximum length (red line), thickness (blue line) of fabella, and the distance between the fabella and the insertion of the lateral head of the gastrocnemius onto the femur (green line); **b** Axial MRI of the knee shows the measurement of the maximum width of the fabella (yellow line); MRI of the knee show (**c**) concave and (**d**) flat contours of the articulating groove of the lateral femoral condyle of the femur (arrow)

In patients where a fabella was present, two readers (one radiologist with 4-year-experience and one orthopedist with 3-year-experience) independently measured the parameters of the fabella, including the maximum length, thickness and width, and distance between the fabella and the insertion of the lateral head of the gastrocnemius onto the femur (DFI), based on MRI films on sagittal, coronal or axial views of the knee [5, 14] (Fig. 2a-b). The intraclass correlation coefficient (ICC) was used to assess the interobserver reliability, while measurements in a random sample of 50 knees were repeated by the radiologist 2 weeks later to assess intraobserver reliability. We chose ICC (2,1) for measurement reliability assessment according to Koo et al. [15]. The presence of any articulating groove was documented, which was defined as either a flat or concave contour of the lateral femoral condyle (Fig. 2c-d). The definition and measurements were described previously [14]. The length/thickness ratio, width/thickness ratio and length/width ratio of fabellae were calculated to present their morphology.

### Statistics

Statistical analyses were performed by SPSS 26.0 (SPSS Inc., Chicago, IL, USA). Continuous variables were stated as the mean ± standard deviation (SD), and categorical variables were stated as percentages and frequency distributions. To assess intergroup differences, the independent samples t test was used for continuous variables showing a normal distribution, the Mann–Whitney U test for nonnormally distributed variables, the chi-square test for categorical variables, and Fisher’s exact test for those with small sample sizes. To assess multigroup differences with heterogeneity of variance, the Kruskal–Wallis H test was used. The Spearman nonparametric correlation test was used for the correlation analysis. Odds ratios (ORs) and 95% confidence intervals (CIs) were calculated to investigate whether sex, knee side, age and fabellar presence were related to meniscal tears or ligament injuries. Multiple logistic regression analysis was used to estimate adjusted ORs and 95% CIs of whether factors were related to meniscus tears or ligament injuries. For each parameter, the receiver operating characteristic (ROC) curve and the area under the curve (AUC) and its 95% CI were calculated. The AUC was tested by the z test with a significance level of 0.5. Indices whose AUCs differed significantly from 0.5 were included in a logistic regression model. The maximal Youden index was used to determine the optimal cutoff value. In the case of multiple cutoff values, the diagnostic OR was used. Accordingly, there was a unique cutoff value for every index. The diagnostic performance of the indices was analyzed by calculations including AUC, Youden index, cutoff value, sensitivity, specificity, accuracy and diagnostic OR via logistic regression model and chi-square test. All of the statistical tests were 2-sided, and *P* values < 0.05 were considered statistically significant if not stated otherwise.

## Results

### Patients and MRI measurements

A total of 1011 knees from 979 patients aged 8 to 91 years (41.47 ± 15.39, mean ± SD, years) were included (Appendix Table A2), of which 46 had ACL rupture, 12 had PCL rupture, 183 had MM tears, 110 had LM tears and 709 had none of the above. Of the included patients, 527 right knees and 484 left ones were examined. To determine whether fabella prevalence is higher in knees with MM tears than in those without MM tears, the power of 1011 knees post hoc was calculated. As a result, our sample size could reach 1-beta > 0.99, and when alpha = 0.05, the power sufficient.

The excellent reliability of the MRI measurements was established with a minimum ICC of 0.851 (95% CI 0.822–0.876) for inter-observer reliability, and 0.880 (95% CI 0.797–0.930) for intra-observer reliability (Appendix Table A3).

### Fabellar prevalence and parameters

FThe overall fabellar prevalence was 39.8% (402/1011 knees) (Table 1). A total of 179/402 (44.5%) fabellae had articulating grooves, 50/179 (27.9%) had concave grooves, and the other 129/179 (72.1%) had flat grooves. Overall, the length, thickness, width and DFI of the 402 fabellae are shown in Appendix Table A4. The size of the fabellae in men was found to be consistently larger, and the DFI was longer than that in women (all *p* < 0.001). However, there was no significant difference in fabellar parameters between the knee sides. Generally, the size of fabellae with articulating grooves was larger than that without articulating grooves (all *p* < 0.001). Analysis of the size of the fabellae according to age groups showed that the length (*p* < 0.001) and thickness (*p* = 0.011) of fabellae and DFI were significantly different (Appendix Table A4 ).

**Table 1:** The Fabellar prevalence ^a^

	Absent	Present	*P* value
Gender			
Male	265 (57.0)	200 (43.0)	0.051
Female	344 (63.0)	202 (37.0)	
Side			
Right	303 (57.5)	224 (42.5)	0.063
Left	306 (62.3)	178 (36.8)	
ACL rupture			
Yes	30 (65.2)	16 (34.8)	0.658
No	579 (60.0)	386 (40.0)	
PCL rupture			
Yes	6 (50.0)	6 (50.0)	0.466
No	603 (60.4)	396 (39.6)	
MM tear			
Yes	61 (33.3)	122 (66.7)	< 0.001
No	548 (66.2)	280 (33.8)	
LM tear			
Yes	63 (57.3)	47 (42.7)	0.507
No	545 (60.6)	355 (39.4)	
Age group^b^			
≤ 20	58 (75.3)	19 (24.7)	< 0.001
21–30	136 (71.6)	54 (28.4)	
31–40	174 (65.7)	91 (34.3)	
41–50	114 (58.2)	82 (41.8)	
51–60	77 (57.5)	57 (42.5)	
≥ 61	50 (33.6)	99 (66.4)	
Overall	609 (60.2)	402 (39.8)	-

^a^Values are presented as n (%). *P* values refer to chi-square test for pooled values. *ACL* anterior cruciate ligament, *PCL* posterior cruciate ligament, *MM* medial meniscus, *LM* lateral meniscus

^b^r = 0.237, *P* < 0.001, Spearman nonparametric correlation test

The length/thickness ratio and width/thickness ratio were found to be significantly different between sexes, ages and articulating groove conditions but not between knee sides (Table 2). Specifically, those ratios showed a moderate correlation with age (r = 0.463, *p* < 0.001; *r* = 0.303, *p* < 0.001), which increased from young to old age groups in patients older than 20 years.

**Table 2:** The Fabellar parameters ^a^

Fabellar parameter	Length, mm	Thickness, mm	Width, mm	DFI, mm	Length/Thickness	Width/Thickness	Length/Width
Gender							
Male (n = 202)	7.96 ± 1.80	5.00 ± 1.61	6.86 ± 2.05	34.8 ± 4.5	1.68 ± 0.42	1.44 ± 0.43	1.22 ± 0.31
Female (n = 200)	7.08 ± 2.37	4.06 ± 1.31	6.08 ± 2.37	31.3 ± 3.9	1.81 ± 0.51	1.54 ± 0.57	1.23 ± 0.31
*P* value	< 0.001	< 0.001	< 0.001	< 0.001	0.015	0.044	0.528
Side							
Right (n = 224)	7.55 ± 2.33	4.53 ± 1.59	6.41 ± 2.40	33.0 ± 4.7	1.74 ± 0.47	1.46 ± 0.44	1.24 ± 0.32
Left (n = 178)	7.49 ± 1.90	4.52 ± 1.47	6.54 ± 2.05	33.0 ± 4.4	1.75 ± 0.47	1.53 ± 0.58	1.20 ± 0.30
*P* value	0.781	0.972	0.336	0.956	0.856	0.395	0.367
Articulating groove							
Yes (n = 179)	8.68 ± 1.85	5.44 ± 1.41	7.36 ± 2.12	32.8 ± 4.6	1.67 ± 0.44	1.39 ± 0.37	1.24 ± 0.30
No (n = 223)	6.59 ± 1.92	3.79 ± 1.21	5.75 ± 2.09	33.2 ± 4.5	1.81 ± 0.48	1.58 ± 0.58	1.21 ± 0.32
*P* value	< 0.001	< 0.001	< 0.001	0.349	< 0.001	< 0.001	0.381
MM tear							
Yes (n = 122)	8.22 ± 2.36	4.38 ± 1.45	7.11 ± 2.37	32.6 ± 4.3	1.97 ± 0.50	1.70 ± 0.60	1.21 ± 0.33
No (n = 280)	7.21 ± 1.98	4.59 ± 1.58	6.19 ± 2.14	33.2 ± 4.7	1.65 ± 0.42	1.40 ± 0.43	1.23 ± 0.30
*P* value	< 0.001	0.294	0.001	0.314	< 0.001	< 0.001	0.408
Age group^b^							
≤ 20 (n = 19)	7.79 ± 2.18	5.29 ± 1.42	6.64 ± 1.59	33.5 ± 5.8	1.49 ± 0.29	1.33 ± 0.40	1.20 ± 0.32
21–30 (n = 54)	7.17 ± 1.97	5.08 ± 1.76	6.42 ± 2.15	34.0 ± 4.8	1.47 ± 0.33	1.32 ± 0.39	1.16 ± 0.27
31–40 (n = 91)	6.92 ± 1.77	4.61 ± 1.58	6.07 ± 2.06	33.4 ± 4.3	1.56 ± 0.32	1.35 ± 0.36	1.20 ± 0.29
41–50 (n = 82)	7.02 ± 1.83	4.29 ± 1.48	6.09 ± 2.32	33.6 ± 5.0	1.71 ± 0.37	1.43 ± 0.44	1.23 ± 0.30
51–60 (n = 57)	7.63 ± 2.10	4.33 ± 1.43	6.65 ± 2.28	32.6 ± 4.4	1.86 ± 0.52	1.61 ± 0.55	1.21 ± 0.32
≥ 61 (n = 99)	8.55 ± 2.45	4.30 ± 1.40	7.03 ± 2.47	31.8 ± 4.0	2.07 ± 0.52	1.71 ± 0.65	1.28 ± 0.35
*P* value	< 0.001	0.011	0.078	0.115	< 0.001	< 0.001	0.417
Overall (n = 402)	7.52 ± 2.15	4.53 ± 1.54	6,47 ± 2.25	33.0 ± 4.6	1.75 ± 0.47	1.49 ± 0.51	1.22 ± 0.31

^a^Values are presented as mean ± standard deviation mm. *P* values refer to independent samples t test, Mann–Whitney U test or Kruskal–Wallis H test for pooled values. *DFI* distance between the fabella and the insertion of the lateral head of the gastrocnemius onto the femur, *MM* medial meniscus. ^b^r = 0.463, *P* < 0.001 for length/thickness ratio, r = 0.303, *P* < 0.001 for width/thickness ratio; Spearman nonparametric correlation test

### Fabellar presence and MM tear

For knee injuries, no significant difference was observed in fabellar prevalence in knees with or without ACL rupture, PCL rupture or LM tears (Table 1). However, the fabellar prevalence was 66.7% in knees with MM tears, which was higher than the prevalence in knees without MM tears (33.8%, p < 0.001). The association was found in all parts (anterior, middle, posterior) of MM, as well as in younger patients (age < 50 years) and in older patients (age ≥ 50 years) (Table 3). The presence of fabella was found in relation to MM tears in subgroups, in male (*p* < 0.001) and female (*P* < 0.001) patients, in right (*p* < 0.001) and left (p < 0.001) knees, and in all age groups, except for patients under 20 years (Appendix Table A5). Since fabellar presence was more frequent in knees with MM tears, the univariate OR of 3.914 (95% CI 2.788–5.496) was calculated (Table 4). Age was also found to be a risk factor after logistic regression. Multiple logistic regression analysis revealed that fabellar presence and age were predictive for MM tears. The adjusted OR indicated that MM tears increased with fabellar presence and from young to old age groups (Table 4).

**Table 3:** Subgroup analysis of association between fabella and MM tear ^a^

Subgroup	MM tear	Absent	Present	*P* value
MM tear location ^b^				
Anterior	Yes	6 (18.2)	27 (81.8)	< 0.001
	No	603 (61.7)	375 (38.3)	
Middle	Yes	40 (31.5)	87 (68.5)	< 0.001
	No	569 (64.4)	315 (35.6)	
Posterior	Yes	55 (33.7)	108 (66.3)	< 0.001
	No	554 (65.3)	294 (34.7)	
Age				
< 50	Yes	22 (39.3)	34 (60.7)	< 0.001
	No	453 (65.9)	204 (31.1)	
≥ 50	Yes	39 (31.7)	88 (69.3)	< 0.001
	No	95 (55.6)	76 (44.4)	

^a^Values are presented as n (%). *P* values refer to chi-square test for pooled values. *MM* medial meniscus. ^b^One knee may present more than one location of MM tear

**Table 4:** Odds ratios of risk factors for medial meniscal tear ^a^

		Coefficient		Standard Error		df		*P* value		Odds Ratio		95% Confidence Interval
Univariate Odds Ratio												
Gender												
Male vs Female		0.221		0.165		1		0.181		1.248		0.902–1.725
Side												
Right vs Left		-0.070		0.164		1		0.670		0.933		0.677–1.285
Fabella												
Present vs Absent		1.366		0.173		1		< 0.001		3.914		2.788–5.496
Age		0.80		0.07		1		< 0.001		1.084		1.069–1.098
Age > 40												
Yes vs No		2.250		0.223		1		< 0.001		9.489		6.124–14.702
Age group												
≤ 20						5		< 0.001				
21–30		0.623		0.794		1		0.432		1.865		0.394–8.835
31–40		0.811		0.764		1		0.289		2.250		0.503–10.062
41–50		2.098		0.740		1		0.005		8.152		1.910–34.790
51–60		2.942		0.739		1		< 0.001		18.961		4.451–80.775
≥ 61		3.691		0.735		1		< 0.001		40.104		9.479–169.361
Multivariate Odds Ratios												
Fabellar presence												
Yes vs No	0.989		0.190		1		< 0.001		2.690		1.853–3.903	
Age	0.074		0.007		1		< 0.001		1.077		1.063–1.091	
Intercept	-5.468		0.364		1		< 0.001		0.004		n/a	

^a^Factors with an increased unvariate odds ratios of risk were included in the logistic regression model. *n/a* not applicable

### Fabellar parameters and MM tear

**Fig. 3:**
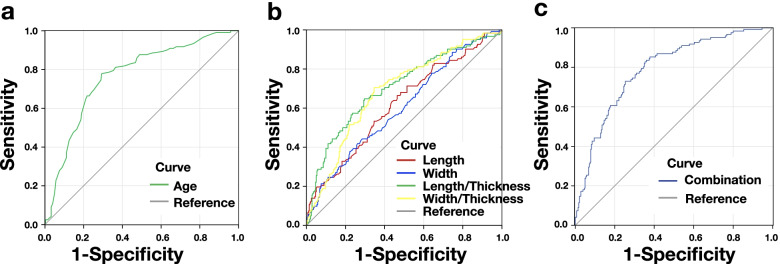
Receiver operator characteristic curve analysis. **a** Age yielded an area under the curve (AUC) of 0.766; **b** Fabellar parameters whose AUC differed significantly from 0.5 were showed. Length (red line, AUC = 0.609), width (blue line, AUC = 0.604), length/thickness ratio (green line, AUC = 0.706), width/thickness ratio (yellow line, AUC = 0.688). **c** Combination = width + length/thickness + age (AUC = 0.791). Reference line (grey line): AUC = 0.5

In 402 patients with fabellae, the fabellar parameters of knees with or without MM tears were compared. (Appendix Table A6). The length (*p* < 0.001) and width (*p* = 0.001) of fabellae, but not thickness or DFI, were significantly larger in knees with MM tears than in those without MM tears. The length/thickness ratio (*p* < 0.001) and width/thickness ratio (*p* < 0.001) were larger in knees with MM tears than in those without MM tears, but no significant difference in the length/width ratio was found (Table 2). Subgroup analysis showed similar patterns in both gender, knee side, articulating groove subgroup and elderly patients (> 40 years) The highest AUC among fabellar parameters was reported for the length/thickness ratio (0.706; 95% CI, 0.649–0.763), with a sensitivity of 64.8% and specificity of 70.4% to predict MM tears (Table 5, Fig. 3, Appendix Tables A7 and A8). The calculated cutoff of 1.776 (Youden index = 0.706) was associated with an increased risk for MM tears (OR = 4.361, 95% CI 2.777–6.848) and allowed an accurate allocation to knees with MM tears versus those without MM tears in 69.4% of the cases. The combination via logistic regression resulted in an AUC of 0.791 (*p* < 0.001, OR = 7.939, 95% CI 4.094–12.853), which was higher than the length/thickness ratio alone with an AUC of 0.706 (*p* = 0.024; Table 5). However, the AUC of the combination was not significantly different from that with age, with an AUC of 0.766 (Table 5).

**Table 5:** Diagnostic performance among age and Fabellar parameters ^a^

	Length	Width	Length/Thickness	Width/Thickness	Age	Combination^b^
Sensitivity	0.664	0.418	0.648	0.705	0.779	0.730
Specificity	0.536	0.739	0.704	0.641	0.707	0.746
Accuracy	0.559	0.681	0.694	0.653	0.748	0.741
DOR	2.280	2.037	4.361	4.507	8.496	7.939
AUC	0.619	0.604	0.706	0.688	0.766	0.791
AUC: 95% CI	0.559–0.679	0.544–0.664	0.649–0.763	0.633–0.744	0.716–0.816	0.744–0.837
*P* for AUC = 0.5^c^	0.001	0.012	< 0.001	< 0.001	< 0.001	< 0.001
*P* for AUC = Combination^d^	< 0.001	< 0.001	0.024	0.005	0.473	n/a

^a^*DFI* distance between the fabella and the insertion of the lateral head of the gastrocnemius onto the femur, *DOR* diagnostic odds ratio, *AUC* area under curve, *CI* confidence interval, *n/a* not applicable. ^b^Combination = Width + Length/Thickness + Age, Logistic regression: Backward. ^c^*P* value of each AUC tested against 0.5; binomial *z* test. ^d^*P* value of each AUC tested against the AUC of combination; binomial *z* test

## Discussion

The findings showed a relatively high prevalence (39.8%) in the Chinese population and demonstrated that fabella presence and parameters are related to MM tears. The fabellar parameters varied among the patient gender, age, articulating groove, and MM tear conditions but not between the sides of the legs. Multivariate analysis showed that both fabellar presence and age were risk factors for MM tears, which indicated that the fabella should not be identified as a simple normal anatomic variant. A prediction model combining age, width, and length/thickness ratio showed good diagnostic performance with an AUC of 0.741 and might aid clinicians in identifying patients at risk for an MM tear and informing patients for their higher MM tear risk.

### Fabellar prevalence and parameters varies

Fabellar prevalence ranges among ethnic groups, e.g., from 3.1% to 31.3% in Caucasian populations and from 30.6% to 92% in Asian populations [2–9, 11–14, 16–25]. Our study findings were consistent with the relatively high prevalence reported in the Asian population [2, 5, 6, 8, 9, 12, 14, 17, 22]. A previous study suggested that the form of the fabella was genetically controlled; however, related genes and pathways were not identified [16]. The ossification of fabellae was correlated with environmental factors, such as mechanical stimuli [6], which was supported by an Asian lifestyle, including kneeling, squatting and tailor sitting. All of the above factors lead to persistent pressure on the fabellae [5, 14].

A controversial relationship was observed between age and fabellar prevalence. Most studies have reported that age is not correlated with fabellar prevalence [2, 3, 13, 14, 16, 21, 22]. However, a positive correlation between age and fabellar presence was observed in our study, which was similar to a large sample size study reported by Hou et al. [8]. These controversial results were assumed to be attributed to the small sample size, which might not be powerful enough to detect such relationships, and the radiological methods they used, which could not distinguish cartilaginous fabellae.

For the fabella size, larger dimensions were recorded in males than in females, as reported previously [14, 18]. In our study, 44.5% of fabellae had articular grooves on the lateral femoral condyle, which was less than that in previous studies [7, 14]; however, we agreed with the fact that a fabella with an articular groove was more likely to be larger [14]. These findings supported that larger fabellae might be more efficient in shifting load through surrounding structures [14, 18]. The fabellar length and thickness varied among age groups, and a trend for this pattern was discovered for width; however, no consistent correlation between age and fabellar parameters was found in our study. To assess the fabellar morphology, the fabellar length/thickness ratio, width/thickness ratio and length/width ratio were calculated. We found that the smaller the fabellae, the larger the length/thickness ratio and width/thickness ratio, i.e., the flatter the fabellae, which reflected the biomechanical advantage of larger fabellae [14, 18]. Our results further suggested that larger fabellae could be tougher against persistent pressure to maintain their original oval-shaped morphology Most previous fabella studies only estimated the prevalence of fabella, and studies with fabella measurements are limited. Only two studies [14, 22] measured the length, thickness and width of the fabella. Chew et al. [14] measured the fabella size of 80 patients who underwent arthroscopy and reported that the average length, thickness and width were 7.06 mm, 4.89 mm and 6.12 mm, respectively. Tabira et al. [22] measured the fabella size of 51 cadavers and reported that the length, thickness and width were 8.2 mm, 5.4 mm, and 9.9 mm, respectively. The size of the fabella measured by Tabira et al. [22] was larger than that measured by Chew et al. [14] and the present study. This may be attributed to the older age of the patients (74.5 vs. 28.4, 41.5 years) in the latter two studies. This result was also supported by our correlation analysis, which indicated that elderly individuals might have larger fabella. The DFI measured by Chew et al. [14] was 33.2 mm, which was supported by our relatively larger sample size (33.0 mm). The fabellar length/thickness ratio, width/thickness ratio and length/width ratio were only calculated in the present study. We further found that the length/thickness ratio and width/thickness ratio were correlated with MM tears. Considering the potential clinical significance of fabellar morphology, future fabella studies should report these measurements to allow further biodynamic research.

### Fabella and MM tear

Previous studies have suggested that fabellae were associated with medical conditions, such as osteoarthritis [8, 26], although none of them considered fabellae as an influencing factor for knee ligaments or meniscus disorders. The present study first revealed that fabellar presence and morphology were associated with MM tears. However, the causal biodynamic mechanism needs to be confirmed by experimental study. Age was a main confounding factor in this study. Although degeneration led to both the presence of fabellae and MM tears [8, 24], we found more fabellae in knees with MM tears among patients older than 20 years. Further logistic regression showed that fabellar presence was an independent risk factor for MM tears, and an ROC analysis demonstrated that fabellar parameters influenced the predictive model, indicating that mechanisms other than age-related degeneration contributed to the association between fabellae and MM tears. A radiographic analysis considering the presence and severity of osteoarthritis might provide a chance to draw a more robust conclusion. In contrast, the OPL was a relatively consistent structure on the posterior aspect of the knee. The OPL originates from the posterior surface of the posteromedial tibia condyle, merges with fibers from the semimembranosus tendon and from the posteromedial part of the capsule, then converges and courses in a diagonal oblique course, and finally attaches to the fabella when that is present [27, 28]. As a structure involved in both posteromedial and posterolateral corners [29], the OPL plays a role in preventing excessive external rotation and extension of the knee [12, 28]. The fabella is considered a stabilizer during this procedure [7]. For this purpose, forces might shift from the fabella along the OPL and separate into a horizontal force and a vertical force [28]; then, it could make a persistent stretch upon the fabella. As a result, its length and width are extended, and thus, a flatter morphology is shown. On the other hand, an equal but opposite force chronically influences the posteromedial corner, thereby leading to posteromedial corner disorders, which decrease the dynamic function of the MM and increase the risk of injury [24]. This hypothesis might explain the flatter fabella seen in knees with MM tears. In knees where a fabella is absent, OPL attaches to the tendon of the lateral head of the gastrocnemius [28]. Without forces transferred from the fabella, the OPL might sustain less force as a dynamic knee stabilizer. Further experimental analysis is needed to determine the causal relationship between the fabella and MM tears, especially the effect of OPL and other surrounding structures on the development and degeneration of the fabella.

### Clinical relevance

Meniscus injury is a common disease in arthroscopic practice [30]. Meniscus tears are the most serious type of meniscus injury. Arthroscopic meniscectomy or meniscal repair is the most recommended surgery for patients with degenerative meniscal tears or traumatic meniscal tears. Our study demonstrated that for a patient with knee pain, the patient is more likely to have an MM tear if the patient has a fabella. In clinical settings, radiography is often the first radiologic examination for patients with knee pain. If the X-ray shows that the patient has a fabella, or even a flat fabella, the patient is recommended to undergo further MRI examinations to determine whether they have an MM tear, which potentially needs arthroscopic surgery.

Okano et al. [31] described a patient who experienced posterolateral knee pain after total knee arthroplasty due to fabella and cured the patient with fabellectomy. Although fabella syndrome could be cured with fabellectomy, as Dekker et al. [26] presented recently, preoperative planning is encouraged, especially in patients with posterolateral knee pain, which includes acquiring a detailed history, assessing the symptoms, performing specific tests concerning fabellar lesions, and radiological evaluation [8]. Intraoperatively, assessment of fabellar impingement against surrounding structures is needed to avoid postsurgical complications. Patients' complaints about posterolateral knee pain should be taken seriously as a sign of potential biomechanical imbalance other than normal postoperative phenomena, even in a patient who underwent medial meniscectomy. These considerations might help clinicians determine whether the fabella should be treated.

### Limitations

There are several limitations in our study. First, our cross-sectional study design was unable to make causal inferences, and only patients with knee pain were included in our study. Moreover, detailed information, such as body mass index, career, habits and anatomic risk factors, was not available for analysis due to the retrospective nature. An external validation may provide more reliable measurements of the model performance. Second, plain films were not available for all included patients. However, MRI measurements are sensitive enough for the detection of fabella [5, 8], and more adequate for detection of MM tears in this study. Third, our model was built mainly based on MRI measurements without clinical risk factors for MM tears. The model would be more practicable if clinical risk factors were included. Fourth, our study did not provide suggestions on whether patients need to be surgically treated. A cohort study with follow-up may provide insights for clinical decision-making. Last, our study did not provide experimental evidence for a causal relation between fabella and MM tears. 

## Conclusion

The presence and morphology of the fabella are significantly associated with an increased risk for MM tears. This might be underestimated and neglected in diagnostic workups before. Clinicians should focus more on MM tears in patients with fabella and with flatter fabella.

## Supplementary Information

.

**Additional file 1. **

## Data Availability

The datasets used and/or analyzed during the current study are available from the corresponding author on reasonable request.

## Abbreviations

ACL: Anterior cruciate ligament
AUC: The receiver operating characteristic curve and the area under the curve
CI: Confidence interval
DFI: Distance between the fabella and the insertion of the lateral head of the gastrocnemius onto the femur
FFL: Fabellofibular ligament
ICC: Intraclass correlation coefficient
LM: Lateral meniscus
MM: Medial meniscus
MRI: Magnetic resonance imaging
OPL: Oblique popliteal ligament
OR: Odds ratios
PCL: Posterior cruciate ligament
ROC: Receiver operating characteristic
SD: Standard deviation
