# Supplementary material for: The prevalence and parameters of fabella and its association with medial meniscal tear in China: a retrospective study of 1011 knees
Source: BMC Musculoskelet Disord. 2022 Mar 1;23:188. doi: 10.1186/s12891-022-05092-4 (PMC8886965; doi:10.1186/s12891-022-05092-4)
Supplement: Supplementary file 1 — Additional file 1. [file 12891_2022_5092_MOESM1_ESM.docx]

**Appendix Material for “The prevalence and parameters of fabella and its association with medial meniscal tear in China: a retrospective study of 1011 knees”**

Appendix Detailed exclusion criteria.

(a) postsurgical scans: (1) medical records of knee surgery (n = 16); (2) detectable implants on MRI (n = 131).

(b) difficult to discriminate the fabella and posterior osteophytes or loose bodies: experienced radiologist (with more than 20 years’ experience) and orthopedist (with more than 20 years’ experience) could not discriminate (n = 50).

(c) conditions not suitable for measurement:

- knee malalignment: (1) medical records of knee malalignment; (2) detectable narrowing of medial or lateral tibiofemoral joint space on coronal view of MRI. (n = 0. The patients were asked to underwent plain radiography of whole lower limb in our institution. They usually did not undergo knee MRI.)
- osteoarthritis: (1) medical records of osteoarthritis; (2) typical appearance of severe osteoarthritis on MRI confirmed by radiologist. (n = 0. The patients with severe osteoarthritis are usually excluded due to “difficult to discriminate the fabella and posterior osteophytes or loose bodies”.)
- acute injury: (1) acute knee dislocation (n = 20); (2) acute dislocated fracture (n = 34)
- gouty arthritis: (1) medical records of gouty arthritis (n = 2); (2) typical appearance of gouty arthritis supported by blood uric acid test (n = 5).
- rheumatic arthritis: (1) medical records of rheumatic arthritis (n = 0); (2) typical appearance of rheumatic arthritis supported by autoimmune test (n = 1).
- synovitis: (1) medical records of synovitis (n = 2); (2) typical appearance of synovitis confirmed by radiologist (n = 9).
- tumors: (1) medical records of tumors in knee; (2) detectable tumor on knee MRI (n = 33. Our institution is one of the most significant MSK center in China; therefore, many bone tumor patients come to us for further treatments.)

(d) poor image quality or nonstandard posture: decided by radiologist (n = 9).

Appendix Table A1 Pilot study ^a^

|  | Absent | Present | P value |
| --- | --- | --- | --- |
| Gender |  |  |  |
| Male | 19 | 15 | 0.577 |
| Female | 33 | 33 |  |
| Side |  |  |  |
| Right | 35 | 35 | 0.541 |
| Left | 17 | 13 |  |
| ACL rupture |  |  |  |
| Yes | 4 | 2 | 0.458 |
| No | 48 | 46 |  |
| PCL rupture |  |  |  |
| Yes | 0 | 1 | 0.296 |
| No | 52 | 47 |  |
| MM tear |  |  |  |
| Yes | 14 | 22 | 0.049 |
| No | 38 | 26 |  |
| LM tear |  |  |  |
| Yes | 6 | 7 | 0.651 |
| No | 46 | 41 |  |
| Overall | 52 | 48 | - |

^a^ The fabellar prevalence are presented as n; *P* values refer to chi-square test for pooled values. *ACL* anterior cruciate ligament, *PCL* posterior cruciate ligament, *MM* medial meniscus, *LM* lateral meniscus.

Appendix Table A2 Age, Gender and Knee Side Examined in Patients ^a^

|  | Age, mean ±standard deviation, y | Male sex, n (%) | Right Knee, n (%) |
| --- | --- | --- | --- |
| Fabella | | | |
| Present (n = 402) | 38.44 ± 14.28 | 202 (50.2) | 224 (55.7) |
| Absent (n = 609) | 46.07 ± 15.88 | 265 (43.5) | 303 (49.8) |
| *P* value | < 0.001 | 0.051 | 0.063 |
| Medial Meniscal Tear | | | |
| Yes (n = 183) | 55.31 ± 13.23 | 76 (41.5) | 98 (53.6) |
| No (n = 828) | 38.42 ± 14.11 | 389 (47.0) | 429 (51.8) |
| *P* value | < 0.001 | 0.181 | 0.670 |
| Overall | 41.47 ± 15.39 | 465 (46.0) | 527 (52.1) |

^a^ *P* values refer to independent samples t test, Mann-Whitney U test or chi-square test for pooled values.

Appendix Table A3 Inter- and Intraobserver Reliability Among Fabellar Parameters ^a^

| Parameter | Interobserver Reliability (n = 402) | | *P* value | Intraobserver Reliability (n = 50) | | *P* value |
| --- | --- | --- | --- | --- | --- | --- |
|  | ICC | 95%CI |  | ICC | 95%CI |  |
| Length | 0.934 | 0.920-0.945 | < 0.001 | 0.987 | 0.977-0.993 | < 0.001 |
| Thickness | 0.949 | 0.938-0.958 | < 0.001 | 0.985 | 0.947-0.991 | < 0.001 |
| Width | 0.959 | 0.951-0.966 | < 0.001 | 0.988 | 0.985-0.990 | < 0.001 |
| Length/Thickness | 0.873 | 0.787-0.925 | < 0.001 | 0.949 | 0.912-0.971 | < 0.001 |
| Width/Thickness | 0.905 | 0.886-0.921 | < 0.001 | 0.973 | 0.953-0.985 | < 0.001 |
| Length/Width | 0.858 | 0.829-0.881 | < 0.001 | 0.974 | 0.955-0.985 | < 0.001 |
| DFI | 0.851 | 0.822-0.876 | < 0.001 | 0.880 | 0.797-0.930 | < 0.001 |

^a^ *ICC* intraclass correlation coefficient, *CI* confidence interval, *DFI* distance between the fabella and the insertion of the lateral head of the gastrocnemius onto the femur.

Appendix Table A4 The Fabellar Parameters ^a^

|  | Length, mm | Thickness, mm | Width, mm | DFI, mm | Length/Thickness | Width/Thickness | Length/Width |
| --- | --- | --- | --- | --- | --- | --- | --- |
| Gender | | | | | | | |
| Male  (n = 202) | 7.96 ± 1.80 (3.19-12.76) | 5.00 ± 1.61 (1.78-11.05) | 6.86 ± 2.05 (2.72-13.12) | 34.8 ± 4.5 (24.8-45.5) | 1.68 ± 0.42 (0.66-3.65) | 1.44 ± 0.43 (0.65-4.05) | 1.22 ± 0.31 (0.66-2.72) |
| Female  (n = 200) | 7.08 ± 2.37 (2.19-15.47) | 4.06 ± 1.31 (1.56-8.94) | 6.08 ± 2.37 (1.68-13.44) | 31.3 ± 3.9 (22.4-43.0) | 1.81 ± 0.51 (0.74-3.83) | 1.54 ± 0.57 (0.53-5.71) | 1.23 ± 0.31 (0.54-2.48) |
| Statics | U=14384.5 | U=13273.5 | U=14479.5 | t’=-8.288 | U=17359.5 | U=17854.0 | U=19465.0 |
| *P* value | p<0.001 | p<0.001 | p<0.001 | p<0.001 | p=0.015 | p=0.044 | p=0.528 |
| Side | | | | | | | |
| Right  (n = 224) | 7.55 ± 2.33 (2.19-15.47) | 4.53 ± 1.59 (1.56-11.05) | 6.41 ± 2.40 (1.68-13.12) | 33.0 ± 4.7 (22.4-45.5) | 1.74 ± 0.47 (0.74-3.83) | 1.46 ± 0.44 (0.53-3.00) | 1.24 ± 0.32 (0.64-2.71) |
| Left  (n = 178) | 7.49 ± 1.90 (3.22-13.81) | 4.52 ± 1.47 (1.59-8.52) | 6.54 ± 2.05 (2.06-13.44) | 33.0 ± 4.4 (24.8-45.3) | 1.75 ± 0.47 (0.66-3.11) | 1.53 ± 0.58 (0.65-5.71) | 1.20 ± 0.30 (0.54-2.48) |
| Statics | t’=-0.278 | U=19895.5 | U=18823.0 | U=19872.0 | U=19726.0 | U=18591.5 | U=18893.0 |
| *P* value | p=0.781 | p=0.972 | p=0.336 | p=0.956 | p=0.856 | p=0.395 | p=0.367 |
| Articulating groove | | | | | | | |
| Yes  (n = 179) | 8.68 ± 1.85 (4.93-15.47) | 5.44 ± 1.41 (2.51-11.05) | 7.36 ± 2.12 (3.16-13.12) | 32.8 ± 4.6 (22.4-45.5) | 1.67 ± 0.44 (0.85-3.14) | 1.39 ± 0.37 (0.69-2.79) | 1.24 ± 0.30 (0.60-2.48) |
| No  (n = 223) | 6.59 ± 1.92 (2.19-12.75) | 3.79 ± 1.21 (1.56-8.94) | 5.75 ± 2.09 (1.68-13.44) | 33.2 ± 4.5 (22.6-44.1) | 1.81 ± 0.48 (0.66-3.83) | 1.58 ± 0.58 (0.53-5.71) | 1.21 ± 0.32 (0.54-2.72) |
| Statics | U=8308.5 | U=7076.0 | U=11416.0 | U=18874.5 | U=15838.5 | U=15882.5 | U=18944.5 |
| *P* value | p<0.001 | p<0.001 | p<0.001 | p=0.349 | p<0.001 | p<0.001 | p=0.381 |
| MM tear | | | | | | | |
| Yes  (n = 122) | 8.22 ± 2.36 (2.67-15.47) | 4.38 ± 1.45 (1.78-8.94) | 7.11 ± 2.37 (2.34-13.44) | 32.6 ± 4.3 (22.6-42.7) | 1.97 ± 0.50 (0.66-3.14) | 1.70 ± 0.60 (0.53-5.71) | 1.21 ± 0.33 (0.54-2.71) |
| No  (n = 280) | 7.21 ± 1.98 (2.19-14.30) | 4.59 ± 1.58 (1.56-11.05) | 6.19 ± 2.14 (1.68-13.12) | 33.2 ± 4.7 (22.4-45.5) | 1.65 ± 0.42 (0.74-3.83) | 1.40 ± 0.43 (0.62-4.05) | 1.23 ± 0.30 (0.64-2.40) |
| Statics | U=13015.5 | U=15956.0 | U=13529.0 | U=16001.5 | U=10042.0 | U=10644.0 | U=16194.5 |
| *P* value | p<0.001 | p=0.294 | p=0.001 | p=0.314 | p<0.001 | p<0.001 | p=0.408 |
| Age group ^b^ | | | | | | | |
| ≤20  (n = 19) | 7.79 ± 2.18 (3.66-11.94) | 5.29 ± 1.42 (2.19-7.90) | 6.64 ± 1.59 (4.00-9.49) | 33.5 ± 5.8 (25.8-42.5) | 1.49 ± 0.29 (1.08-2.10) | 1.33 ± 0.40 (0.78-2.07) | 1.20 ± 0.32 (0.64-1.75) |
| 21-30  (n = 54) | 7.17 ± 1.97 (2.85-11.82) | 5.08 ± 1.76 (2.19-8.91) | 6.42 ± 2.15 (2.69-13.12) | 34.0 ± 4.8 (25.6-43.5) | 1.47 ± 0.33 (0.74-2.48) | 1.32 ± 0.39 (0.69-2.50) | 1.16 ± 0.27 (0.74-1.94) |
| 31-40  (n = 91) | 6.92 ± 1.77 (2.19-12.29) | 4.61 ± 1.58 (1.68-11.05) | 6.07 ± 2.06 (1.68-11.66) | 33.4 ± 4.3 (25.6-44.1) | 1.56 ± 0.32 (0.66-2.75) | 1.35 ± 0.36 (0.62-2.31) | 1.20 ± 0.29 (0.65-2.14) |
| 41-50  (n = 82) | 7.02 ± 1.83 (3.32-11.60) | 4.29 ± 1.48 (1.56-8.07) | 6.09 ± 2.32 (2.06-12.35) | 33.6 ± 5.0 (22.4-45.3) | 1.71 ± 0.37 (1.04-2.80) | 1.43 ± 0.44 (0.84-2.68) | 1.23 ± 0.30 (0.73-2.00) |
| 51-60  (n = 57) | 7.63 ± 2.10 (3.22-12.76) | 4.33 ± 1.43 (1.78-7.84) | 6.65 ± 2.28 (3.00-12.91) | 32.6 ± 4.4 (24.0-43.0) | 1.86 ± 0.52 (0.85-3.64) | 1.61 ± 0.55 (0.79-4.04) | 1.21 ± 0.32 (0.60-2.48) |
| ≥61  (n = 99) | 8.55 ± 2.45 (2.67-15.47) | 4.30 ± 1.40 (2.06-8.94) | 7.03 ± 2.47 (2.34-13.44) | 31.8 ± 4.0 (22.6-45.5) | 2.07 ± 0.52 (1.02-3.83) | 1.71 ± 0.65 (0.53-5.71) | 1.28 ± 0.35 (0.54-2.72) |
| Stastics | H=31.233 | H=14.825 | H=9.901 | H=8.852 | H=87.285 | H=37.839 | H=4.987 |
| *P* value | p<0.001 | p=0.011 | p=0.078 | p=0.115 | p<0.001 | p<0.001 | p=0.417 |
| Overall  (n = 402) | 7.52 ± 2.15 (2.19-15.47) | 4.53 ± 1.54 (1.56-11.05) | 6,47 ± 2.25 (1.68-13.44) | 33.0 ± 4.6 (22.4-45.5) | 1.75 ± 0.47 (0.66-3.83) | 1.49 ± 0.51 (0.53-5.71) | 1.22 ± 0.31 (0.54-2.72) |

^a^Values are presented as mean ± standard deviation (range). *P* values refer to independent samples t test, Mann-Whitney U test or Kruskal-Wallis H test for pooled values. *DFI* distance between the fabella and the insertion of the lateral head of the gastrocnemius onto the femur, *MM* medial meniscus.

^b^r=.463, P<.001 for length/thickness ratio, r=.303, P<.001 for width/thickness ratio; Spearman nonparametric correlation test.

Appendix Table A5 Prevalence of Fabella and Medial Meniscal Tear ^a^

|  | Medial Meniscal Tear | | χ2 | *P* value | OR | 95%CI | Fabellar Prevalence | Medial Meniscal Tear Prevalence |
| --- | --- | --- | --- | --- | --- | --- | --- | --- |
|  | Yes | No |  |  |  |  |  |  |
| Gender |  |  |  |  |  |  |  |  |
| Male (n = 202) | 49 (64.5) | 151 (38.8) | 17.074 | <0.001 | 2.860 | 1.714-4.774 | 200 (43.0) | 76 (16.3) |
| Female (n = 200) | 73 (68.2) | 129 (29.4) | 55.677 | <0.001 | 5.160 | 3.271-8.139 | 202 (37.0) | 107 (19.6) |
| Side |  |  |  |  |  |  |  |  |
| Right (n = 224) | 65 (66.3) | 159 (37.1) | 27.955 | <0.001 | 3.345 | 2.106-5.312 | 224 (42.5) | 98 (18.6) |
| Left (n = 178) | 57 (67.1) | 121 (30.3) | 40.664 | <0.001 | 4.677 | 2.836-7.712 | 178 (36.8) | 85 (17.6) |
| Age ≤ 40 ^b^ | | | | | | | | |
| Yes (n = 164) | 17 (65.4) | 147 (29.1) | 15.309 | <0.001 | 4.613 | 2.011-10.584 | 164 (30.8) | 26 (4.9) |
| No (n = 238) | 105 (66.9) | 133 (55.9) | 27.613 | <0.001 | 2.869 | 1.924-4.279 | 238 (49.7) | 157 (32.8) |
| Age group |  |  |  |  |  |  |  |  |
| ≤20 (n = 19) | 1 (50.0) | 18 (24.0) |  | 0.435 | 3.167 | 0.188-53.233 | 19 (24.7) | 2 (1.1) |
| 21-30 (n = 54) | 7 (77.8) | 47 (26.0) | 8.910 | 0.003 | 9.979 | 2.002-49.733 | 54 (28.4) | 9 (4.7) |
| 31-40 (n = 91) | 9 (60.0) | 82 (32.8) | 4.643 | 0.031 | 3.073 | 1.058-8.925 | 91 (34.3) | 15 (5.7) |
| 41-50 (n = 82) | 20 (57.1) | 62 (38.5) | 4.102 | 0.043 | 2.129 | 1.015-4.466 | 82 (41.8) | 35 (17.9) |
| 51-60 (n = 57) | 27 (60.0) | 30 (33.7) | 8.453 | 0.004 | 2.950 | 1.406-6.188 | 57 (42.5) | 45 (33.6) |
| ≥61 (n = 99) | 58 (75.3) | 41 (56.9) | 5.638 | 0.018 | 2.308 | 1.149-4.635 | 99 (66.4) | 77 (51.7) |
| Overall (n = 402) | 122 (66.7) | 280 (33.8) | 67.525 | <0.001 | 3.914 | 2.788-5.496 | 402 (39.8) | 183 (18.1) |

^a^ Values are presented as n (%). *P* values refer to chi-square test or Fisher exact test for pooled values. *OR* odds ratio, *CI* confidence interval.

^b^ The mean age of participants is 41.47 years; therefore, we selected 40 as the cutoff.

Appendix Table A6 Parameters of Fabella and Medial Meniscal Tear among Subgruops ^a^

|  | Length, mm | Thickness, mm | Width, mm | DFI, mm | Length/Thickness | Width/Thickness | Length/Width |
| --- | --- | --- | --- | --- | --- | --- | --- |
| Gender | | | | | | | |
| Male (n = 202) | 0.012 | 0.171 | 0.039 | 0.363 | <0.001 | <0.001 | 0.637 |
| Female (n = 200) | <0.001 | 0.386 | 0.001 | 0.433 | <0.001 | <0.001 | 0.440 |
| Side | | | | | | | |
| Right (n = 224) | 0.012 | 0.922 | 0.014 | 0.794 | <0.001 | <0.001 | 0.308 |
| Left (n = 178) | 0.003 | 0.153 | 0.023 | 0.214 | <0.001 | <0.001 | 0.989 |
| Articulating groove | | | | | | | |
| Yes (n = 179) | 0.001 | 0.006 | 0.006 | 0.082 | <0.001 | <0.001 | 0.949 |
| No (n = 223) | 0.023 | 0.663 | 0.031 | 0.636 | <0.001 | 0.001 | 0.271 |
| Age ≤ 40 | | | | | | | |
| Yes (n = 164) | 0.292 | 0.859 | 0.514 | 0.878 | 0.083 | 0.132 | 0.987 |
| No (n = 238) | 0.005 | 0.830 | 0.003 | 0.681 | <0.001 | <0.001 | 0.096 |
| Age group | | | | | | | |
| ≤ 20 (n = 19) | 0.100 | 0.855 | 0.100 | 0.361 | 0.144 | 0.465 | 1.000 |
| 21-30 (n = 54) | 0.928 | 0.146 | 0.990 | 0.625 | 0.021 | 0.041 | 0.990 |
| 31-40 (n = 91) | 0.273 | 0.315 | 0.599 | 0.312 | 0.936 | 0.979 | 0.968 |
| 41-50 (n = 82) | 0.216 | 0.434 | 0.078 | 0.746 | 0.746 | 0.170 | 0.103 |
| 51-60 (n = 57) | 0.955 | 0.786 | 0.603 | 0.817 | 0.330 | 0.231 | 0.284 |
| ≥ 61 (n = 99) | 0.093 | 0.963 | 0.061 | 0.757 | 0.008 | 0.006 | 0.386 |
| Overall (n = 402) | <0.001 | 0.294 | 0.001 | 0.314 | <0.001 | <0.001 | 0.408 |

^a^ *P* values refer to independent samples t test or Mann-Whitney U test comparing fabellar parameters between knees with and without medial meniscal tear.

Appendix Table A7 Determination of Cutoff ^a^

|  | Length, mm | Thickness, mm | Width 1, mm | Width 2, mm | DFI, mm | Length/Thickness | Width/Thickness | Length/Width | Age | Combination 1 ^b^ | Combination 2 ^b^ | Combination 3 ^b^ |
| --- | --- | --- | --- | --- | --- | --- | --- | --- | --- | --- | --- | --- |
| Sensitivity | 0.664 | 0.689 | 0.443 | 0.418 | 0.590 | 0.648 | 0.705 | 0.975 | 0.779 | 0.852 | 0.730 | 0.844 |
| Specificity | 0.536 | 0.343 | 0.714 | 0.739 | 0.483 | 0.704 | 0.641 | 0.068 | 0.707 | 0.632 | 0.746 | 0.636 |
| Youden Index | 0.200 | 0.032 | 0.157 | 0.157 | 0.019 | 0.706 | 0.688 | - | 0.748 | 0.699 | 0.741 | 0.699 |
| Accuracy | 0.575 | 0.448 | 0.632 | 0.681 | 0.515 | 0.352 | 0.359 | 0.043 | 0.486 | 0.484 | 0.476 | 0.480 |
| AUC | 0.609 | 0.467 | 0.604 | 0.604 | 0.468 | 0.706 | 0.688 | 0.474 | 0.766 | 0.790 | 0.791 | 0.790 |
| AUC: 95% CI | 0.559-0.679 | 0.407-0.527 | 0.544-0.664 | 0.544-0.664 | 0.408-0.528 | 0.649-0.763 | 0.633-0.744 | 0.413-0.535 | 0.716-0.816 | 0.743-0.837 | 0.744-0.837 | 0.743-0.837 |
| *P* value for AUC =0.5 ^c^ | 0.001 | 0.294 | 0.012 | 0.012 | 0.314 | < 0.001 | < 0.001 | 0.408 | < 0.001 | < 0.001 | < 0.001 | < 0.001 |
| Cutoff ^d^ | 7.420 | 3.725 | 7.150 | 7.405 | 31.75 | 1.776 | 1.453 | 0.831 | 47.5 | n/a | n/a | n/a |
| DOR | 2.280 | n/a | 1.985 | 2.037^d^ | n/a | 4.361 | 4.507 | n/a | 8.496 | 9.929 | 7.939 | 9.460 |
| DOR: 95% CI | 1.464-3.550 | n/a | 1.277-3.087 | 1.301-3.188 | n/a | 2.777-6.848 | 2.844-7.142 | n/a | 5.158-13.993 | 5.693-6.625 | 4.094-12.852 | 5.477-16.341 |
| *P* value for Χ^2^ | < 0.001 | n/a | 0.002 | 0.002 | n/a | < 0.001 | < 0.001 | n/a | < 0.001 | < 0.001 | < 0.001 | < 0.001 |
| *Kappa* for Age ^e^ | 0.159 | n/a | n/a | 0.140 | n/a | 0.404 | 0.276 | n/a | n/a | 0.872 | 0.801 | 0.792 |
| *Kappa* for Length/Thickness ^e^ | 0.148 | n/a | n/a | 0.022 | n/a | n/a | 0.428 | n/a | 0.404 | 0.392 | 0.430 | 0.420 |

^a^ *DFI* distance between the fabella and the insertion of the lateral head of the gastrocnemius onto the femur, *DOR* diagnostic odds ratio, *AUC* area under curve, *CI* confidence interval, *n/a* not applicable.

^b^ Combination 1 = Length + Width/Thickness + Age, Logistic regression: Forward; Combination 2 = Width + Length/Thickness + Age, Logistic regression: Backward; Combination 3 = Length + Width/Thickness + Width + Length/Thickness + Age, Logistic regression: Enter

^c^ *P* value of each AUC tested against 0.5; binomial *z* test.

^d^ Cutoff determined by DOR.

^e^*P* value of the difference among the AUC age, length/thickness and each index; chi-square test.

Appendix Table A8 Multivariate Odds Ratios of Fabellar Parameters for Medial Meniscal Tear

|  | Coefficient | Standard Error | df | *P* value | Multivariate Odds Ratios | 95% Confidence Interval |
| --- | --- | --- | --- | --- | --- | --- |
| Logistic regression: Forward | | | | | | |
| Age | 0.057 | 0.009 | 1 | <0.001 | 1.059 | 1.040-1.077 |
| Length | 0.132 | 0.060 | 1 | 0.027 | 1.141 | 1.015-1.283 |
| Width |  |  | 1 | 0.713 |  |  |
| Length/Thickness |  |  | 1 | 0.562 |  |  |
| Width/Thickness | 0.785 | 0.268 | 1 | 0.003 | 2.193 | 1.298-3.707 |
| Intercept | -5.834 | 0.678 | 1 | <0.001 |  |  |
| Logistic regression: Backward | | | | | | |
| Age | 0.055 | 0.009 | 1 | <0.001 | 1.057 | 1.038-1.076 |
| Length |  |  | 1 | 0.792 |  |  |
| Width | 0.157 | 0.055 | 1 | 0.004 | 1.169 | 1.050-1.302 |
| Length/Thickness | 0.772 | 0.284 | 1 | 0.007 | 2.164 | 1.241-3.773 |
| Width/Thickness |  |  | 1 | 0.684 |  |  |
| Intercept | -5.967 | 0.690 | 1 | <0.001 |  |  |
| Logistic regression: Enter | | | | | | |
| Age | 0.056 | 0.009 | 1 | <0.001 | 1.057 | 1.038-1.077 |
| Length | 0.050 | 0.210 | 1 | 0.811 | 1.052 | 0.697-1.587 |
| Width | 0.088 | 0.232 | 1 | 0.795 | 1.092 | 0.693-1.721 |
| Length/Thickness | 0.490 | 0.830 | 1 | 0.555 | 1.632 | 0.321-8.306 |
| Width/Thickness | 0.352 | 0.904 | 1 | 0.697 | 1.422 | 0.242-8.368 |
| Intercept | -5.935 | 0.695 | 1 | <0.001 | 1.057 |  |
